# Safety hazards in abdominal surgery related to communication between surgical and anesthesia unit personnel found in a Swedish nationwide survey

**DOI:** 10.1186/s13037-015-0089-y

**Published:** 2016-01-13

**Authors:** Katarina Göransson, Johan Lundberg, Olle Ljungqvist, Elisabet Ohlsson, Gabriel Sandblom

**Affiliations:** Department of Intensive Care and Perioperative Medicine, Skåne University Hospital, Lund, Sweden; Dept of Surgery, Faculty of Medicine and Health, Örebro University, Örebro, Sweden; Department of Anesthesiology and Intensive Care, Sahlgrenska University Hospital, Göteborg, Sweden; Center for Digestive Diseases, Karolinska Institutet, Karolinska University Hospital, SE-141 86 Stockholm, Sweden

## Abstract

**Background:**

Many adverse events occur due to poor communication between surgical and anesthesia unit personnel. The aim of this study was to identify strategies to reduce risks unveiled by a national survey on patient safety.

**Methods:**

During 2011–2015, specially trained survey teams visited the surgery departments at Swedish hospitals and documented routines concerning safety in abdominal surgery. The reports from the first seventeen visits were reviewed by an independent group in order to extract findings related to routines in communication between anesthesia and surgical unit personnel.

**Results:**

In general, routines regarding preoperative risk assessment were safe and well- coordinated. On the other hand, routines regarding medication prior to surgery, reporting between the different units, and systems for reporting and providing feedback on adverse events were poor or missing. Strategies with highest priority include: 1. a uniform national health declaration form; 2. consistent use of admission notes; 3. systems for documenting all important medical information, that is accessible to everyone; 4. a multidisciplinary forum for the evaluation of high-risk patients; 5. weekly and daily scheduling of surgical programs; 6. application of the WHO check list; 7. open dialog during surgery; 8. reporting based on SBAR; 9. oral and written reports from the surgeon to the postoperative unit; and 10. combined mortality and morbidity conferences.

**Conclusion:**

One repeatedly occurring hazard endangering patient safety was related to communication between surgical and anesthesia unit personnel. Strategies to reduce this hazard are suggested, but further research is required to test their effectiveness.

## Background

Although surgical care has gradually become more specialized, technically advanced, and focused on efficacy, it has not eliminated adverse events due to human errors. In the United States, adverse events are estimated to occur in 2.9 % to 3.7 % of all hospitalizations [1]. Surgery includes many hazardous procedures where the risk of committing a mistake is high. In Sweden, data from the Swedish Patient Insurance (Landstingens Ömsesidiga Försäkringsbolag; LÖF) show that in 2012, reported mistakes resulting in adverse events increased by 7 % to 13 905 events, and specialties such as general surgery and orthopaedics accounted for the majority of mistakes.

Nagpal et al. analysed the entire surgical process from pre-operative assessment to post-operative care and the subsequent transfer to the wards [2]. Mistakes related to communication were seen in pre-, peri- as well as post-operative phases, but were the most common during pre-operative assessment, in most cases because of forgetfulness and ignorance, but also because of uncertainty in the distribution of responsibilities and the hierarchy. Mistakes in the post-operative phase were also common, including missing information and excess of it. In interviews with the concerned staff, distracting stimuli in the environment, lack of standardized communication routines and primitive systems for exchange of information were identified as the main causes of mistakes [3]. In another study, the quality of reporting by anaesthesiologists was considered adequate in only 32 % of the cases. Despite the fact that in 96 % of the cases the patients handed over to post-operative care were stable, nurses in the post-operative unit were satisfied with the transfer reports in only 48 % of the cases [4]. Greenberg et al. found that most misunderstandings involved verbal communication between a single reporter and a recipient [5], particularly if a surgeon was involved.

Many mistakes are also made in theatre. By tradition, the staff working in theatre represents several disciplines, which makes communication more complicated [6]. Effective teamwork and communication are crucial to safety in the operating theatre, but communication is often complicated by differences in professional practices across disciplines and the ways in which professionals collaborate and may also be impaired by differences in language, attitude, and the level of education. Loyalty to the discipline may be greater than that to the team and to the common task at hand. Several studies have shown how the difficulties that arise when information is perceived differently by various members of the team cause tension, conflicts, and practical problems. Social barriers in the form of gender, ethnic background, and socio-economic differences may add to the divergent perception of information by different members of a team [7].

The present study therefore sought to analyse the reports from a national survey on patient safety to find out the ten most frequent hazards related to communications between anaesthetists and surgical unit personnel and to assess possible strategies to reduce the risks from such miscommunication.

## Methods

### Survey organization

To survey the routine procedures followed in Swedish surgical practice and to recommend any adaptations to the routine suited to each individual unit, a nationwide programme was started in 2011, titled Safe Abdominal Surgery. The programme offered every surgical unit in Sweden a visit by a survey team including 4–5 representatives from surgery and anaesthesiology. The programme focused exclusively on abdominal surgery; vascular, urological, and gynaecological surgeries were not included. The teams consisted of nurses and physicians who had undergone specialized training.

**Fig. 1:**
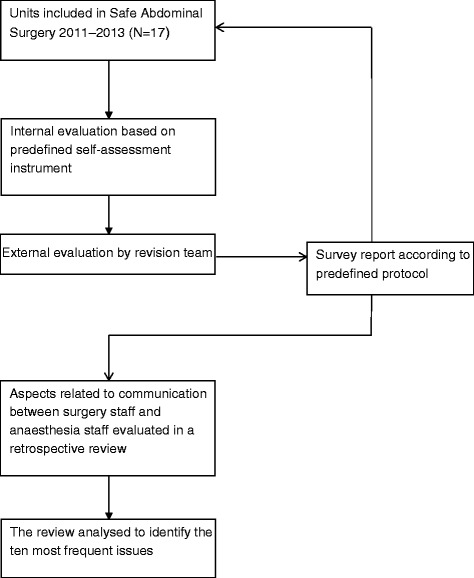
Flow chart of the revisions process and assessment of the outcome

Altogether 56 units in Sweden were invited to participate in the programme. By the end of 2015, 43 of them will have been visited by a survey team. Written consent was obtained from all participating units prior to the revision. To start with, the participating units were requested to fill in a self-assessment instrument comprising 24 items on measures considered important to preventing adverse events in abdominal surgery (Fig. 1). The self-assessment instrument is presented in Appendix 1.

Data on admissions at each unit during 2012 were obtained from Statistics Sweden [8].

Following the self-assessment, each unit was visited by a survey team consisting of 4–5 members, representing both nurses and physicians from an independent unit, who had attended a two-day training programme. The training programme included an introduction in the aims and methods of the survey. The self-assessments of the units were also evaluated during the introduction.

The visits were based on interviews with personnel from different categories, including representatives from surgical and anaesthesiology units (residents and specialists in surgery and anaesthesiology). Nurses from the outpatient clinic and surgical wards and anaesthesia and surgery personnel from the operations room (OR) and post-operative care facility were also interviewed about the routines followed at their respective units. The head of the surgical department was responsible for assembling representatives from all personnel categories. Furthermore, the survey teams were instructed to address as many of the co-workers as possible in order to obtain information from unprepared staff members.

The observations and outcomes of the interviews were recorded in a standardized protocol based on the same items as those presented in Appendix 1. The report specified a certain number of routines that needed improvement at each unit.

### Review of the revision reports

A preliminary retrospective review of the survey reports pointed to a repeating pattern of problems in communication between the surgical staff and the personnel from anaesthesia. To examine these observations more systematically, a group was commissioned by the Patient Insurance LÖF [9] to evaluate the reports and to enumerate all the problems related to patient safety in the routines involving communications between the surgical staff and the personnel from anaesthesia. This group was also multi-professional, comprising nurses and physicians from surgical and anaesthesia units.

The present study is based on the observations and conclusions from the survey teams. The reports compiled between 2011 and 2013 from 17 surgical units were reviewed, and the ten most frequent hazards related to communications between personnel of the surgical and the anaesthesia units were selected for retrospective evaluation. Each of these hazards was subdivided into 1–6 issues, and safety systems and routines at each of the units were assessed based on these issues.

Adverse events were defined as unintended experiences related to a surgical procedure. Risks were defined as potential sources of adverse events that could have been avoided by using better routines. Safety hazards were defined as potential sources of all adverse events, both avoidable and unavoidable. Mistakes were defined as adverse events due to inappropriate method of care and, therefore, potentially preventable. As the survey did not involve any new intervention or other interference with the health care, no application to ethical committee was sent.

### Data from the review reports

The reports were assessed using a predefined template with the following questions. The questions were in Swedish; an English translation is given here.

1. **Is adequate documentation of the patient’s health status obtained prior to surgery?**
  Adequate health declaration form
  Consistent routines for filling in the health declaration form
  System of patients’ records accessible to both surgical and anaesthesia unit personnel
  Routines for documenting health status including information on any communicable agents requiring isolation
2. **Routines for securing correct diagnosis and intended procedure prior to surgery**
  Adequate documentation
  Functioning health records system
3. **Routines for securing pre-operative risk assessment**
  Appropriate American Society of Anesthesiologists (ASA) physical status classification
  Documentation of ASA classification accessible to everyone
  Forum for multidisciplinary evaluation of high-risk patients
  Other risk assessment systems parallel to ASA
  Adequate documentation of all assessments
4. **Is the patient’s status optimal prior to surgery?**
  Routines to ensure that nothing is neglected
5. **Is appropriate medication confirmed prior to surgery and anaesthesia?**
  Documentation available to anaesthesia as well as surgical-unit personnel
6. **Routines for clear peri-operative communication**
  WHO checklist and/or any other system for crew resource management used consistently and correctly
7. **Is peri- and post-operative pain management clearly specified?**
  Clear routines
  Documentation available to anaesthesia as well as surgical-unit personnel
8. **Is exchange of information ensured when patients are transferred from post-operative unit and intensive care unit?**
  Clear and consistent routines defined
9. **Is correct exchange of information ensured between the medical units that a patient passes through during in-patient treatment and the units responsible for the patient after discharge?**
  Documentation available to anaesthesia as well as surgical-unit personnel
  Routines for documenting all measures taken
  Principles of situation background assessment recommendation (SBAR) used consistently in communication between different units
10. **Are there routines for providing feedback on adverse events or systematic safety problems that could be improved?**
  Mortality and morbidity conferences comprising anaesthesia and surgical unit personnel

In the template, each of the issues listed under the subheadings was rated on the following scale:

1. Unsatisfactory
2. Partly fulfilled
3. Satisfactory
4. Not assessable

### Statistical analysis

To account for inter-observer reliability, three randomly selected reports were reviewed by two independent reviewers and, based on these assessments, intra-class correlation coefficients were estimated. Internal consistency between different items for each respective unit was estimated using Cronbach’s alpha. Missing values were replaced with the means of each unit. The number of adverse events reported to the Swedish Patient Insurance as a percentage of total number of admissions was tested for correlation against the mean score to test whether the outcome of the revisions could predict inadequate safety routines.

## Results

**Fig. 2:**
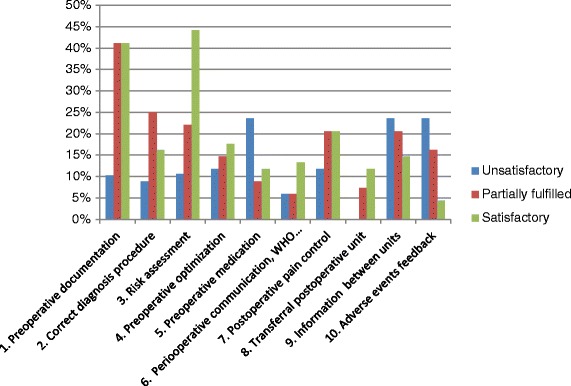
Assessment ratings from 17 survey reports. The figure shows the distributions of all subscales for each issue related to communications between surgical personnel and anaesthesia personnel. Because data were missing for some subscales, the total does not add up to 100 % for each scale. When the outcome was not assessable from the reports, the unit was excluded from the analysis

The results of the 17 survey reports are shown in Fig. 2. The units comprised six university hospitals, four regional hospitals, and seven local hospitals. The median number of admissions during 2012, including admission that did not proceed to surgery, was 22 954 and ranged from 5400 to 105 924. The median number of adverse events reported to the Swedish Patient Insurance during 2007–2014 was 77 and ranged from 23 to 223. Data on adverse events from 2015 are not yet available. The median number of approved reports during the same period was 25 and ranged from 6 to 89.

In general, the routine procedures related to pre-operative risk assessment were safe and well-coordinated. There were, however, some problems in the routines followed during pre-operative medication and those that involved reporting between different units and in the systems for reporting and providing feedback on adverse events. Several survey reports did not provide reliable information on the discharge from post-operative care units and intensive care units: communications between these two units and the receiving units are therefore difficult to evaluate.

In the review reports, a number of problems were mentioned repeatedly (Table 1). Although it is impossible to quantify these observations or to evaluate the effectiveness of the suggested measures to improve safety, some patterns could be identified. The most frequently recorded recommendations are listed in Table 1. The WHO checklist was not used consistently at the units, but it was not possible to determine which of the elements in the checklist were neglected.

**Table 1:** Most frequently reported safety hazards and recommendations to minimize their occurrence

Safety hazards	Recommendations
Unreliable documentation of pre-operative health status	• National uniform health declaration • Standardized surgery notification form (Mandatory registration of the most important data, consistent registration of contagious conditions, and preset limitations of care) • Standardized routines for pre-operative assessment • Routine use of ASA classification • Selective pre-operative anaesthesiologist assessment based on ASA classification (Patients with ASA I-II are assessed on request; those with ASA IV are assessed invariably.)
Divergent systems of documentation between different units	• Consistent admission notes (structured with standard headings, preferably partly delegated to a specialist nurse) • Systems for documenting important medical information accessible to everyone • Systems for patient records with a structure suitable for surgery as well as anaesthesia Harmonization of management programmes of clinics involved in the same course of care • Coordinated and uniform systems for drug prescriptions for anaesthesia and surgery
Insufficient planning of high-risk procedures	• Multidisciplinary forum for evaluation of high-risk patients • Weekly scheduling of surgical programmes operations in the entire unit • Daily scheduling of programmes in each theatre
Inconsistent use of checklists	• Routine use of the WHO checklist
Lack of standardized communication	• Open dialogue during surgery • Team training with anaesthesia and surgery crew • Reporting based on SBAR
No routines for feedback on adverse events	• Mortality and morbidity conferences common to surgery and anaesthesia personnel

Cronbach’s alpha for all items at the 17 units was 0.501, and the intra-class correlation coefficients for the reports assessed by two independent observers are given below.

- Unit 1: 0.441 (95 % confidence interval 0.022–0.728, p = 0.020)
- Unit 2: 0.611 (95 % confidence interval 0.242–0.825, p = 0.002)
- Unit 3: 0.229 (95 % confidence interval −0.227–0.602, p = 0.159)

## Discussion

The nationwide survey showed that in Sweden, the routines for communication between the personnel of anaesthesia units and those of surgical units may involve safety risks. Although efforts have been made in recent years to prevent mistakes in surgical procedures by defining secure routines and by managing the different teams better, many departments continue to lack proper routines for preventing systematic and recurring hazards due to poor communication between the personnel of anaesthesia units and those of surgical units. The recommendations in Table 1 may reduce the risk of adverse events, although further studies are required to prove that the recommendations are effective.

Routine procedures for risk assessment prior to surgery were considered adequate in the majority of units. Precautions to prevent post-operative pain were satisfactory; however, the routines for communication between different departments needed improvement in most of the hospitals. The survey reports did not provide enough data to draw definite conclusions regarding the routines for ensuring correct diagnoses and intended procedures, proper medication and care prior to surgery, peri-operative communication and feedback on adverse events or systematic safety problems; when the reports did provide enough data, the routines for peri-operative medication and interdisciplinary conferences turned out to be either poor or lacking altogether.

Most of the recommendations presented in Table 1 have been evaluated earlier and are now part of safety improvement programmes in many hospitals. Some new and untested routines were also encountered during the review. However, because the main purpose of the study was to identify safety hazards that could be avoided by following well-established measures, only those recommendations that may gain general acceptance are included. Although every survey team included representatives from the fields of surgery and anaesthesia, the mix of anaesthetic nurses, theatre nurses, surgeons, and anaesthesiologists varied from team to team. Since the experiences of these professionals from different fields may have been different, the survey report may also have been influenced by personal views of the team members. Also, even if the standardized protocol was used by all the teams, it was difficult to abstract all data in a uniform way. However, despite the uncertainties related to some issues in the survey reports, the inter-observer reliability, when reviewed retrospectively, was high.

The methods used in the present study were semi-quantitative. The data were derived from a wide range of Swedish surgical units and selection bias cannot be ruled out. The programme was designed to survey Swedish surgical health care in general, which makes it impossible to assess the effectiveness of each of the strategies presented above. Some of the suggested strategies have been tested in larger studies [10, 11], but evidence-based support for these is still very limited. Furthermore, despite firm evidence, there may be reluctance to implement new routines [12]. There was no way of validating the responses in the self-assessment instruments with full security, but at the visits information interviews were undertaken with as many of the staff members as possible, including those were not prepared for the visits in advance.

At the time of the analysis, less than a third of all Swedish surgical units had had participated in the study. More units have participated since. Nevertheless, it is possible that the units that participated during the first three years were those keener than the rest to bring about improvements.

Checklists in medical practice may be an effective way to ensure that no step in a surgical process is missed so as to avoid adverse events that may occur when personnel from two different units – surgery and anaesthesia, for example – are required to work together [13]. The SURPASS checklist accompanies the patient at each stage of the surgical process [14] and is completed by members of the team in the pre-operative ward, the operating theatre, the post-operative unit, and the surgical ward. Another way of improving the surgical process and minimizing the risk of adverse events owing to miscommunication between surgical personnel and personnel of the anaesthesia unit is ERAS, the enhanced-recovery-after-surgery programme. The programme was designed to attenuate the stress response during surgery, mainly by focusing on the need for parenteral analgesia and intravenous fluids. It is based on a checklist and continuous feedback to all personnel on their performance. Adopting the ERAS programme has lowered morbidity by up to 50 % and shortened the surgery-related hospital stay after major abdominal surgery by about 30 % [15–17].

The WHO checklist was created to improve safety in the operating theatre. The purpose of the checklist is to ensure that all conditions are optimal for patient safety and that all the personnel present are identifiable and accountable. The checklist includes 19 items divided into three critical phases of the surgical procedure: before anaesthesia is induced, before the skin incision is made, and before the patient leaves the operating theatre. The results of adopting the checklist have been evaluated in large cohort studies [18, 19] and its use was found to reduce the risk of serious adverse events considerably. Another tool is the SBAR frame (short for situation background assessment recommendation), which is designed to elicit concise and focused information using standardized questions, and can be applied in any situation in which information about a patient is required to be communicated [20].

The present study was performed based on the assumption that checklists improve surgical safety. Although checklists have been reported to reduce hazards [13, 19, 21], they have also been questioned [22]. It is difficult to test safety routines because of the Hawthorne effect, i.e. the very fact that their outcome is going to be recorded may induce the intervention group to perform better. If implemented mechanically, checklists may create a false sense of security and decrease alertness, which can help in dealing with unforeseen hazards. The implementation of checklists should thus be followed by a careful evaluation of their impact on patient safety. The Safe Abdominal Surgery programme, for example, will be evaluated using data on adverse outcomes from gallstone, hernia, and colorectal cancer registries.

## Conclusions

In conclusion, this study shows that in Sweden, many important potential hazards that endanger patient safety in the surgical process are due to miscommunication or poor communication between surgery personnel and those involved in administering anaesthesia. There were problems in the routines followed during pre-operative medication and those that involved reporting between different units and in the systems for reporting and providing feedback on adverse events. These problems may be avoided by consistent use of admission notes, systems for documenting all important medical information, application of the WHO check list; reporting based on SBAR oral and written reports from the surgeon to the postoperative unit; and combined mortality and morbidity conferences. The realization from the present surveymay help to define strategies aimed at reducing the risks from such hazards although further research is required to test the effectiveness of such strategies.

## Appendix 1. Self-assessment instrument

A. Preoperative risk assessment
  1. How do you ascertain that there is a correct diagnose and that the right procedure is planned?
  2. How do you ascertain satisfactory preoperative risk assessment and evaluation?
  3. How do you ascertain an optimal planning prior to surgery
  4. How do you ascertain that all necessary information regarding the health status of the patient is assembled before the procedure?
  5. How do you ascertain that the patient takes active participation in all decisions and that the patient has received all necessary information before surgery?
  6. How do you ascertain that the patient does not carry multiresistant bacteria or other communicable agents?
B. Procedures prior to anesthesia and surgery
  1. How do you ascertain optimal circumstances before surgery?
  2. How do you ascertain adequate peri- and postoperative analgesia
  3. How do you ascertain that antibiotic prophylaxis, thoroboprophylaxis and antiemetics are given according to correct routines?
C. Anesthesia and surgery
  1. How do you ascertain that the right patient undergoes surgery and that the area of surgery is correctly marked?
  2. How do you ascertain that surgical competence is available in case of difficutlies during the procedure?
  3. How do you survey and regulate vital functions during the procedure?
  4. How do you ascertain appropriate surgical environment?
  5. How do you ascertain access to surgical services other than those routinely used?
D. Postoperative Intensive Care Unit and postoperative care
  1. How do you ascertain postoperative monitoring and access to postoperative intensive care?
  2. How do you ascertain appropriate discharge from postoperative care unit?
  3. How do you ascertain monitoring at the ward after discharge from postoperative care unit?
E. Ward, discharge, and outpatient follow-up
  1. How do you ascertain appropriate assessment at discharge?
  2. How do you ascertain that the patient has received and understood all information at discharge?
  3. How do you ascertain that postoperative complications are managed adequately?
F. General aspects
  1. How do you ascertain that basic sanitary routines, including appropriate dressing, is followed during the entire course?
  2. What systems do you have for registering results, adverse events and complications?
  3. How do you ascertain correct exchange of information between the units the patients pass during and after the course of care?
  4. How do you ascertain that all personnel has appropriate knowledge about technical equipment?
  5. Please give a short account of the profile of your unit.
